# Effect of an mHealth Intervention Using a Pedometer App With Full In-Person Counseling on Body Composition of Overweight Adults: Randomized Controlled Weight Loss Trial

**DOI:** 10.2196/16999

**Published:** 2020-05-27

**Authors:** Alberto Hernández-Reyes, Fernando Cámara-Martos, Rafael Molina-Luque, Rafael Moreno-Rojas

**Affiliations:** 1 Universidad de Córdoba Córdoba Spain

**Keywords:** pedometer, physical activity, exercise prescription, diet, health behavior

## Abstract

**Background:**

In clinical practice, it is difficult to convey the benefits of sustained physical activity to adult patients with excess weight or obesity. For this purpose, a goal-setting walking prescription may be an effective strategy.

**Objective:**

This study aimed to determine the efficacy of the intervention of a pedometer app in setting a goal to reach 10,000 steps per day in adults.

**Methods:**

Overweight adults (n=98; mean body mass index 32.53 [SD 4.92] kg/m2) were randomized to one of two conditions (control or intervention). Both groups downloaded a pedometer app that recorded their daily step counts and were given a daily walking goal of 10,000 steps. Subjects participated in a 24-week in-person behavioral weight control program and were asked to monitor their daily levels using the pedometer app. Baseline data were recorded and followed up weekly. Only the intervention group had structured information delivery, a personalized physical activity prescription, and follow-up on number of steps per day.

**Results:**

The results show that regardless of sex or age, prescribing walking increased the number of steps per day by 4806 step on average (standardized β coefficient=–0.813, SE=427.586, *t*=–11.242, *P*<.001).

**Conclusions:**

These results could have implications for improving self-monitoring in overweight adults during periods of weight loss. Health professionals should analyze the implementation of tools that permit them to prescribe, follow up, and encourage the achievement of a goal of physical activity in overweight or obese patients.

**Trial Registration:**

ClinicalTrials.gov NCT03845478; https://clinicaltrials.gov/ct2/show/NCT03845478

## Introduction

Three in four adolescents and one in three adults in the world do not perform at least 30 minutes of physical activity (PA) per day as recommended by the World Health Organization [1]. This represents a severe public health problem, given that physical inactivity is the fourth leading risk factor for global mortality and plays a role in the development of obesity and metabolic syndrome [2,3]. Increasing PA should be a priority in the treatment of weight loss in overweight and obese subjects. Walking is a solution to overcoming physical inactivity [4] due to its low impact, in which the person can control its intensity, duration, and effort in order to reduce the risk of injury [5]. A recent review has deepened the knowledge of the psychosocial factors that increase adherence to a PA protocol, and social support and self-evaluation are presented as critical elements [6]. The prescription of PA by the health professional to sedentary people suffering from obesity, diabetes, or hypertension is defined as the delivery of an individualized exercise prescription for a limited time [6].

Despite efforts in many countries to facilitate access to leisure centers so people can increase PA [7], lack of adherence remains the main problem [8].

Monitoring PA by counting steps per day offers the possibility of standardizing evaluation and follow-up [9]. Although the goal of 10,000 steps a day may not be appropriate for all ages and levels of physical conditioning, it is considered a reasonable and motivating goal for healthy adults, and previous studies have demonstrated its effectiveness in weight loss programs [9,10].

The term mHealth is defined as “public health and medical practices compatible with mobile devices, including smart mobile phones, patient monitoring gadgets, personal digital assistants (PDA), and other wireless devices” [11]. The use of this technology is pervasive, and, in developed countries, the level of penetration reaches almost 100% of the adult population [11]. Mobile apps provide tools, processes, and communications used to support and provide medical care to patients and the general public [12]. Apps monitoring PA are objective and automated, allowing the user to carry a device that tracks their movements [13].

Different methods are available to measure PA, but there is no definite gold standard to measure PA in various clinical settings. Accelerometers and pedometers have been developed for research use because they are easy to wear and portable. Accelerometers can provide quantification and recording of PA [14]. A important issue with accelerometers is how to select cutoff points to define activity intensities. Despite proposed cutoffs for some devices, there is currently no consensus [15], and this inconsistency in the use of accelerometers to delineate exercise makes it difficult to compare findings of different studies [16].

Pedometers are capable of counting the number of steps; they became popular more than a decade ago as a meter gauge and motivator of daily exercise [17]. Their use today has grown significantly thanks to the development of apps capable of collecting and storing information on daily PA concerning walking or running. This technology has been proven to be effective in the strategy to encourage and motivate patients to execute and even increase the number of steps per day [18]. The review by Mansi et al [19] concluded that interventions based on the use of a pedometer were more effective when combined with additional behavioral strategies (eg, setting goals and facilitating access to the information generated).

The main objective of this study was to evaluate the efficacy of prescribing versus recommending PA in a sedentary adult population with overweight or obesity. Another aim of the study was to measure the improvement of body composition in both scenarios. This study sought to extend the findings of Glynn et al [20] by examining the feasibility of this approach of using the Accupedo-Pro pedometer app (Corusen LLC) intervention to promote PA in an overweight adult sample. The hypothesis is that encouraging, following, and involving patients in the use of this app through the establishment of objectives and self-monitoring would significantly increase the number of steps executed per day, thus affecting the amount and quality of weight loss, and we can measure this by comparing total body weight, body fat and overall muscle mass, and body mass index (BMI).

## Methods

### Recruitment

Participants were recruited from private health clinics and sports centers through social network advertisements and direct actions in the centers in the area of Cádiz, Andalusia, Spain. The exclusion and inclusion criteria are listed in Textbox 1. Participants were interested in losing weight and owned a smartphone. Participants attended an orientation session to complete a consent form and baseline questionnaires, including the International Physical Activity Questionnaire and demographic questions. The study protocol complied with the Declaration of Helsinki for medical studies, it was approved by the bioethical committee of Córdoba University and the department of health at the regional government of Andalusia (Act no. 284, ref. 4156), and the study was retrospectively registered with ClinicalTrials.gov [NCT03845478] on February 19, 2019.

**Selection criteria.**

**Inclusion:**

- Aged 18 years and older
- Overweight or obese (body mass index 25 to 49.9 kg/m^2^)
- Own smartphone with Android or iOS operating system and internet access
- Lead sedentary lifestyle
- Have not been on a diet to lose weight within 6 months of the start of the study

**Exclusion:**

- Diabetes treated with oral medications or insulin
- Pregnant
- Chronic renal insufficiency

### Randomization Groups

The number of daily reference steps counted was collected in all the subjects using the Accupedo app for 7 days before randomization. Patients did not receive any information or comments at the time of app installation. Subsequently, subjects were randomly assigned (1:1) using a computerized random number generator, and Accupedo was installed on phones in both groups. In their initial interview, members of the two groups received information on the importance and benefits of walking 10,000 steps a day, but only the intervention group (IG) received follow-up and monitoring to reach this total. Patients in both groups were instructed to use the pedometer daily during all their waking hours. Each week, a member of the research team checked the data on participant apps and recorded the average daily steps taken during the week and month in the computer system. The IG had individualized goal settings, instructions on counting steps for self-assessment, and educational and motivational content to improve self-management. The control group (CG) received a recommendation to count steps, without any reproach in the case of not increasing their PA.

### Push Notifications

Push notifications permit the delivery of timely updates and customized reminders to users. This functionality offers auditory and visual alerts to inform users about an incoming message and invites them to act, even if the app sending the notifications is not currently in use. The methodology we used is explained in greater detail elsewhere [21].

### Outcome Assessments

#### Physical Activity

After randomization, the number of daily steps was averaged each week if the subject’s pedometer was used on three or more days during that week. The difference between the average daily step count and the baseline one was determined for weeks 12 to 24, inclusive. The strata proposed by Tudor-Locke and Bassett were used [9], establishing the following ranges based on the evidence available for classifying PA according to the data provided by the pedometer:

- Fewer than 5000 steps per day can be used as a sedentary lifestyle index
- 5000 to 7499 steps per day is considered not very active
- 7500 to 9999 probably includes some volitional activities (or high demands for occupational activity) and could be considered active
- 10,000 steps or more per day indicates the point used to classify people as quite active

#### Dietary Intervention

The daily energy requirements were determined by estimating the resting energy expenditure using the formula proposed by Harris-Benedict [22]:

- Women: basal metabolic rate = 655.1 + (9.563 × weight in kg) + (1.850 × height in cm) – (4.676 × age in years)
- Men: basal metabolic rate = 66.5 + (13.75 × weight in kg) + (5.003 × height in cm) – (6.755 × age in years)

and multiplying the value obtained by a factor of 1.5 in those patients performing PA [23].

Through 24 weeks, all patients followed a diet with the following allocation of macronutrients: 25% to 30% protein, 40% to 45% carbohydrates, and 30% to 35% fat. The menu was hypocaloric with a reduction of 500 kcal/day during the treatment period to achieve a weekly weight loss of 400 grams. After being included in the study, patients participated in a 1-hour seminar in which the dietitian-nutritionist instructed them on how to make a suitable selection of food. The menu proposed was valid for one week and was given to participants in the weekly revision appointment as the protocol for the next week. The energy and nutritional intake was evaluated by the program Dietowin and the weighing method by Dietowin 8.0 (Dietowin SL) [24].

#### Anthropometrics and Body Composition Measurements

Body fat, muscle mass, and percentage of water, considered as result variables, were monitored by multifrequency electrical impedance (BWB-800A, Tanita Corp), which has been previously validated [25]. This method is based on a 3-compartment model capable of evaluating body fat, muscle mass, and bone mineral content. The independent variables collected were age (years), height (cm), weight (kg), and BMI (kg/m^2^). The anthropometric measurements were taken following the recommendations of the standardized anthropometry handbook [26] by experienced personnel to reduce the coefficient of variation. Each measurement was taken 3 times, and the mean value was calculated. All quantitative variables were measured with the precision of 0.1. A stadiometer (Seca 213, Seca) was employed to measure height.

### Statistical Analysis

#### Power Calculation

A similar previous 6-month trial was used to conduct sample size calculations (α=.05 and power β=80%) based on the expected differences between groups in the use of the pedometer to increase PA and body weight modification in obese adults [27].

#### Statistics

Quantitative variables have been presented with the mean and standard deviation, and qualitative variables in frequencies and percentages. To contrast goodness of fit with a normal distribution of data from quantitative variables, the Kolmogorov-Smirnov test with Lilliefors correction was used. For the bivariate hypothesis, the Student *t* test was performed for 2 means, while for the qualitative variables, the chi-square and Fisher exact tests were employed. For analysis of 3 or more means, analysis of variance of repeated means determined the effects of the intervention at the basal moment at 3 and 6 months, and the correlation between the quantitative variables was verified by the coefficient of Pearson correlation (*r*). Finally, if the normality or homoscedasticity criterion was not met for analysis of variance, the Kruskal-Wallis test was performed. To adjust the possible impact of PA on body composition and its possible role as a confounding factor, adjusted linear regressions were made for each body composition variable (body fat and muscle mass) and weight, calculating the standardized β coefficients. To determine goodness of fit of the models, the standard error, adjusted coefficient of determination, *F* statistic, linearity, and residuals were analyzed. For all statistical analyses, an α error of less than 5% was accepted (*P*<.05) and a 95% confidence interval was calculated. Statistical analysis was performed using SPSS Statistics software version 22.0 (IBM Corp).

## Results

### Characteristics of the Population Studied

**Figure 1:**
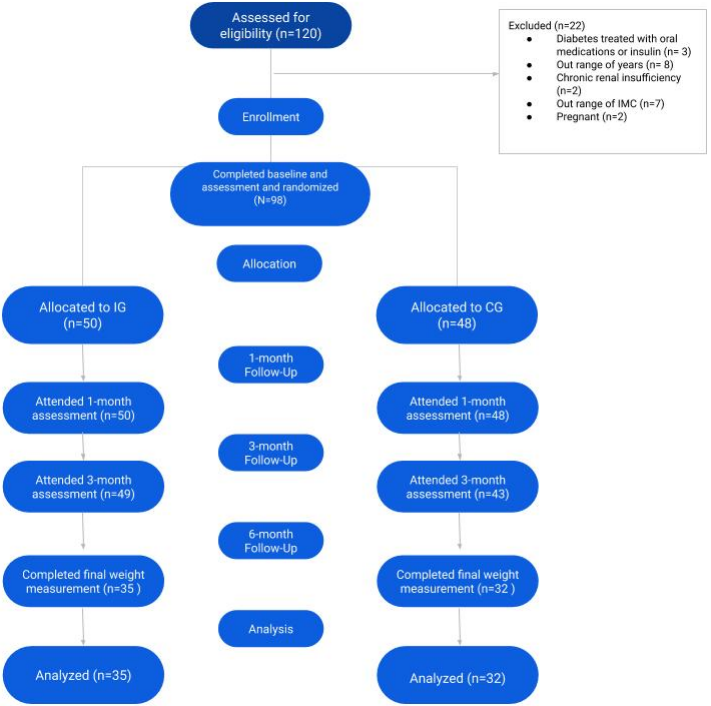
Consolidated Standards of Reporting Trials diagram.

Between January 2016 and December 2018, 98 participants were randomly registered and assigned to the groups (Figure 1). No significant differences were found in the baseline data between the groups, to which the patients were assigned randomly (*P*>.05 for all; Table 1). The attrition rate was 6.12% at 3 months and 31.63% at 6 months and did not differ between groups at either 3 (chi-square = 0.33, *P*=.29) or 6 (chi-square = 0.54, *P*=.09) months.

**Table 1:** Baseline body mass index and demographics of study participants.

Variable		Total (n=67)	CG^a^ (n=32)	IG^b^ (n=35)	*P* value
Age in years, mean (SD)		41.51 (11.29)	42.88 (10.91)	40.26 (11.63)	.35
**Gender, n (%)**					.12
	Female	44 (66)	24 (75)	20 (57)	—
	Male	23 (34)	8 (25)	15 (43)	—
**Education, n (%)**					.24
	Without studies	17 (25)	9 (28)	8 (23)	—
	Ninth grade	25 (37)	15 (47)	10 (29)	—
	High school diploma	10 (15)	3 (9)	7 (20)	—
	University students	15 (22)	5 (16)	10 (29)	—
**Occupation, n (%)**					.01
	Unemployed	4 (6)	1 (3)	3 (9)	—
	Service occupation	32 (48)	18 (56)	14 (40)	—
	Technical, sales, administrative	9 (13)	6 (19)	3 (9)	—
	Executive, professioanl specialty	15 (22)	6 (9)	9 (34)	—
	Retired	4 (6)	4 (13)	0 (0)	—
	Student	3 (5)	0 (0)	3 (9)	—
Initial weight (kg), mean (SD)		—	89.65 (19.32)	91.35 (16.36)	.70
BMI^c^ (kg/m^2^), mean (SD)		—	32.72 (5.56)	32.34 (4.28)	.75
Body fat (%), mean (SD)		—	39.66 (7.59)	36.76 (8.85)	.16
Muscle mass (kg), mean (SD)		—	51.35 (13.38)	54.92 (13.18)	.28
Water (kg), mean (SD)		—	44.10 (4.58)	46.07 (5.66)	.13

^a^CG: control group.

^b^IG: intervention group.

^c^BMI: body mass index.

### Weight Modification and Body Composition Depending on Physical Activity Prescription or Not at 3 and 6 Months

Examining both groups together, participants lost significant weight at both 3 (–6.84 [SD 3.97] kgs; *P*<.001) and 6 months (–7.92 [SD 3.93] kgs; *P*<.001). Weight loss over the study period was significantly different between the groups at 3 and 6 months (Table 2). These results are extrapolated to BMI and loss of total body fat in the cited period. Change in muscle mass was not significant at 3 or 6 months. For body composition, focusing on body fat loss, prescribing PA resulted in losing fat significantly at 3 months (9.56% in IG compared with 6.13% in CG; *P*<.18) and 6 months (15.60% in IG versus 7.04% in CG; *P*<.001).

**Table 2:** Weight loss, body mass index, and body composition of participants.

Variable		CG^a^ (n=32), mean (SD)	IG^b^ (n=35), mean (SD)		*P* value
**Weight change (kg)**					
	3 months	–5.63 (2.60)		–8.06 (2.59)	<.001
	6 months	–6.29 (2.63)		–10.80 (3.31)	<.001
**BMI^c^ change (%)**					
	3 months	–5.91 (2.84)		–8.02 (2.73)	.003
	6 months	–6.32 (2.79)		–10.92 (3.74)	<.001
**Body fat (%)**					
	3 months	37.38 (8.06)		33.24 (8.32)	.02
	6 months	36.94 (7.66)		31.12 (8.62)	<.001
**Muscle mass (kg)**					
	3 months	50.27 (12.78)		53.54 (12.59)	.65
	6 months	50.30 (12.98)		53.44 (12.43)	.64
**Body water (%)**					
	3 months	45.45 (5.26)		48.41 (5.62)	.03
	6 months	46.14 (5.18)		50.07 (5.54)	.005

^a^CG: control group.

^b^IG: intervention group.

^c^BMI: body mass index.

### Increase in Average Number of Steps Depending on the Prescription or Not of Reaching 10,000 Steps a Day

**Figure 2:**
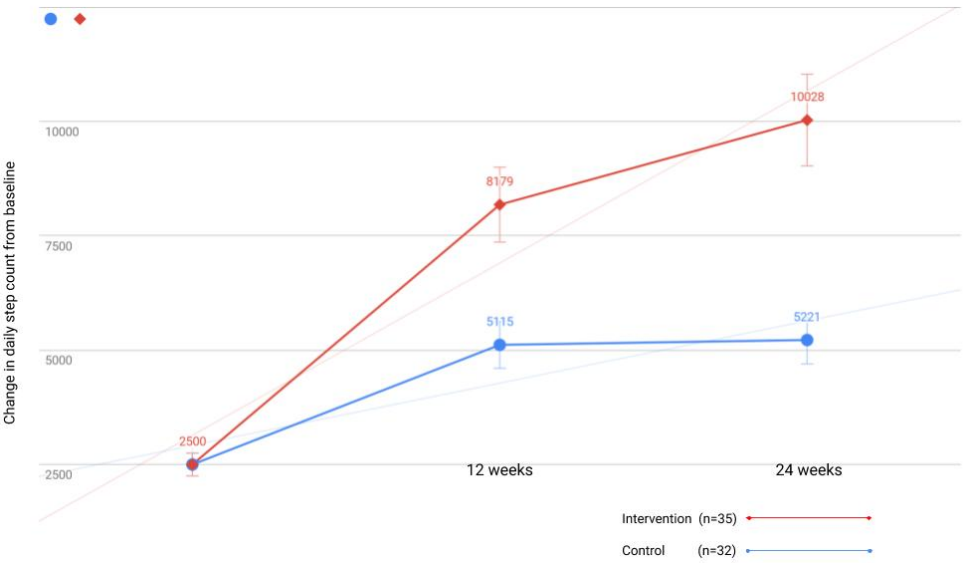
Graph showing interaction effect between time and condition.

Figure 2 illustrates the change for average daily steps from the start of the study to weeks 12 and 24. In week 12, subjects who only followed the recommendation to complete a certain number of steps had an average change from the baseline data of 2615 (SD 1849) steps, compared with an increase of 5679 (SD 4015) steps in the group of patients receiving the prescription. It is interesting to observe that, at 24 weeks, the CG maintained the number of daily steps, with an increase of only 106 (SD 74) steps compared with the IG that achieved 10,028 average daily steps (SD 1307). At 3 months, IG participants recorded an average of 8179.77 (SD 1815.66) steps, which is significantly higher than the average recorded by the patients in the CG (5115.25 [SD 1200.54]) steps (*P*<.001).

### Weight Modification and Body Composition Depending on the Range of Steps Executed

Independently of the intervention or control group, we analyzed the results in weight and body composition according to the range of steps executed. The results (Table 3) show that a higher number of steps resulted in a more significant loss of body weight and BMI at 3 months (*P*<.001) and 6 months (*P*<.001), the highest BMI lost, with identical statistical results.

Focusing again on the results obtained in body fat, we found that, like weight and BMI, the difference was significant for participants in all 4 step ranges (*P*<.001). We observed that in the second period (weeks 13 to 24), patients who walked fewer than 5000 steps per day did not lose additional weight (–4.37 kgs [SD 2.3] in the first 12 weeks and –4.58 [SD 1.75] at 24 weeks). Also, in this range of steps, (<5000 per day), patients in the second period (weeks 13 to 24) began to regain the fat lost in the first 12 weeks (–6.65% [SD 4.54] in the first quarter, and –4.87% [SD 6.13] in the second). This threshold of steps per day is considered to be of concern and might be the reason for not losing weight or fat. The influence of the group assigned was endorsed by the results offered by the linear regression model, adjusted for age and sex (*R*^2^ adjusted=.655, *F*=126.386; *P*<.001). This showed how, regardless of sex and age, being incorporated into the prescription group (standardized β coefficient=–0.813, SE=427.586, *t*=–11.242; *P*<.001) raised the number of steps per day at 6 months to 4806.

**Table 3:** Participant anthropometrics and body composition per step range.

Variable		>10,000 steps, mean (SD)		7500-9999 steps, mean (SD)		5001-7499 steps, mean (SD)		<5000 steps, mean (SD)		*P* value	
**Weight change (kg)**											
	3 months		–9.97 (2.48)		–8.30 (1.93)		–6.61 (2.42)		–4.37 (2.30)		<.001
	6 months		–11.89 (2.88)		–10.51 (3.48)		–7.39 (2.38)		–4.58 (1.75)		<.001
**BMI change (%)**											
	3 months		–10.45 (2.83)		–7.92 (1.58)		–6.62 (2.42)		–4.91 (3.15)		<.001
	6 months		–11.97 (3.59)		–10.80 (3.66)		–7.49 (2.57)		–4.52 (1.70)		<.001
**Body fat (%)**											
	3 months		–15.14 (7.84)		–6.92 (3.44)		–6.83 (5.41)		–6.65 (4.54)		.07
	6 months		–17.13 (9.62)		–14.19 (11.45)		–9.27 (6.03)		–4.87 (6.13)		.97
**Muscle mass (kg)**											
	3 months		–1.51 (4.86)		–3.96 (3.89)		–2.74 (2.95)		–0.08 (3.41)		.08
	6 months		–2.35 (5.11)		–2.45 (4.28)		–2.18 (3.64)		–1.65 (3.78)		.02
**Body water (%)**											
	3 months		9.00 (6.77)		3.29 (3.19)		3.79 (3.31)		2.66 (4.25)		—
	6 months		9.82 (6.51)		9.17 (6.37)		5.52 (6.29)		3.63 (4.03)		.05

^a^CG: control group.

^b^IG: intervention group.

^c^BMI: body mass index.

## Discussion

### Principal Findings

Although performing exercise regularly has been associated with the prevention of a wide range of pathologies in the developed world, the correlation between walking daily and completing a certain number of steps and its percentage quantification in weight loss, fat, and BMI is not clear. This study uses an objective measure of PA through a goal-setting mechanism and its comparison with a control group to elucidate the improvement in body composition in overweight or obese adults.

### Comparison With Prior Work

Our results show that PA prescription, together with the establishment of setting a goal for participants, improves the count of steps at 6 months compared with the use of a pedometer without a goal being fixed. We observed a decrease in the number of average daily steps during the second quarter, after a substantial initial increase. In the CG, a stagnation occurred, while in the IG, it was possible for participants to reach the goal proposed. The reminder messages sent through push notifications and work in the weekly face-to-face consultation increased the effectiveness of Accupedo. The subjects of both groups showed a better commitment, increasing their number of steps. Prescribing versus recommending, self-control, and reinforcement in face-to-face consultations seem to have been effective in promoting and maintaining the number of steps achieved daily. These results in a cohort of patients with obesity referred to a nutrition consultation, in addition to confirming previous findings in studies of similar duration and population, broaden our understanding of the beneficial mechanisms of implementing prescription strategies in a personal consultation with each patient [10,28].

The amount of weight loss in the IG can be considered clinically relevant. Helping subjects establish a realistic goal is a vital part of the success of the program. The motivation to lose weight and adding a component of PA produces a more significant fat loss and BMI adjustment in addition to increasing the degree of adherence [29].

The critical differences between the groups led us to think that, in addition to IG participants burning more calories due to the fact per se of walking more, this stimulus would cause more considerable changes in the individual. With the same diet, walking results in percentage fat losses of 15.40% in the IG compared with 7.65% in the CG. This fact may be due to a significant improvement in the regulation of the energy balance and better general functioning of the organism (ie, precise control of body homeostasis) [30]. It seems logical to propose an active lifestyle as a measure to influence the body composition of overweight or obese subjects, highlighting the impact it can have on the loss of total body fat. The results of our study confirm data obtained previously in which performing moderate PA resulted in weight and fat loss and better body composition of overweight or obese people [31,32].

We found a significant inverse relationship between the number of steps executed and BMI. Our data corroborate those obtained by Wayne et al [33] aimed at the study of this relationship in the young population, as well as those trials on middle-aged people of Thompson et al [34] or elderly ones by Krumm et al [35]. It makes sense that performing a more significant number of steps results in better body composition and BMI. However, we observed diminishing performance over time. When it comes to making a general recommendation regarding the number of steps necessary to reduce body fat and body weight, our results confirm the range proposed by Tudor-Locke [36] (ie, maintaining a level of 8000 to 10,000 steps per day).

Our findings have implications in the field of public health. There is a positive correlation between number of steps taken and amount of body fat loss. Although decrease in BMI can be considered positive, it is a marker that today presents great controversy when interpreting its scope due to the limitations it presents [37]. However, there is consensus on the negative impact that an increase in body fat has on health as it is associated with the risk of cardiovascular disease [38], obesity [39], and an increase in general mortality [40].

Our data on steps counted show the essential objectivity of the established prescription. The review by Kang et al [41] found that pedometer use led to an increase of 2000 to 2500 steps per day, an average figure that coincides with the increment of steps achieved by the CG in our study. However, to reach 10,000 average steps per day, the pedometer per se seems to be ineffective, and some authors have suggested the need to reinforce patient behavior with self-control tools, goal setting, and follow-up to significantly increase the PA performed [42]. We corroborate these results and find them also consistent with those reported by Samdal et al [43], where a definite improvement is observed in the number of steps executed in the IG (through goal setting and in-person counseling) during the first-trimester intervention [43]. We speculate that a period longer than 12 weeks may be necessary for a sedentary and obese person to reach a volume of steps equivalent to 10,000 per day, an aspect that must be taken into account in establishing an objective when prescribing and monitoring PA.

There are several reasons why IG patients lose more weight than CG patients. First, there is self-management, a mechanism that has allowed IG participants to keep a record of self-weighing and monitor themselves to modify their behaviors to achieve goals. Another reason could be motivation and its relationship with behavior change techniques are used to encourage physical activity. The theory of motivation has been defined as being a critical mechanism in the construction process that determines intensity and direction of the action of human behavior [44,45]. The relationship with PA maintenance seemed to be influenced by the presence of an objective establishment and an active follow-up, aspects corroborated in previous trials [46].

### Limitations

Strengths of our study include randomized design, weekly control of PA in all subjects in a face-to-face consultation, demographic data, and the study of body composition. Due to the voluntary nature of the study, a limitation could be the possible self-selection occurring for highly motivated populations. We have been able to improve the degree of adherence to the prescription of PA, but we understand that 6 months, although it is a prudential period, cannot be considered definitive to confirm a change in behavior and that more time is necessary in order to confirm the effectiveness of the prescription. In addition, a 25% attrition rate was expected at 6 months, and the study saw attrition of 30%. While attrition was greater that intended, it was similar to what has been observed in other weight loss interventions [25,47]. No differential attrition rates were observed between groups in our study.

### Conclusion

Establishing goal-setting and feedback mechanisms on PA can increase the effectiveness of prescribing it in people who are overweight or obese. We are aware that the recommendations of the official bodies, the World Health Organization among others, to do PA are not fulfilled, and that people’s sedentary lifestyle is a real problem. Establishing achievable objectives and developing a monitoring system capable of tracking PA and thus being able to help change sedentary habits is feasible and could be more so with the help of technology. The ease of use of the pedometer installed in the smartphone makes this tool an ally for the health care professional, who can, at no cost, have access to patient mobility data on a day-to-day basis. The application of these measures in groups of patients can be investigated in future studies. We have found advantages in short-term adherence (the first 6 months) to PA in patients with a prescription, but we do not know what happens when they leave this follow-up. Studies are needed to determine if the behavioral change persists with the passage of time or patients return to sedentary habits once they have no professional follow-up.

## Appendix

Multimedia Appendix 1 CONSORT-eHEALTH checklist (V 1.6.1).

## Abbreviations

BMI: body mass index
CG: control group
IG: intervention group
mHealth: mobile health
PA: physical activity
